# Supplementary material for: Connecting Histopathology Imaging and Proteomics in Kidney Cancer through Machine Learning
Source: J Clin Med. 2019 Sep 25;8(10):1535. doi: 10.3390/jcm8101535 (PMC6832975; doi:10.3390/jcm8101535)
Supplement: Supplementary file 1 [file jcm-08-01535-s001.zip › SuppFigures-Revised.docx]

Connecting Pathology Imaging and Proteomics in Kidney Cancer through Machine Learning

Francisco Azuaje ^1,*^, Sang-Yoon Kim ^1^, Daniel Perez Hernandez ^1^ and Gunnar Dittmar ^1^

^1^ Quantitative Biology Unit, Luxembourg Institute of Health, Strassen, Luxembourg.

***** Correspondence: francisco.azuaje@lih.lu

**Supplementary Figures**

**
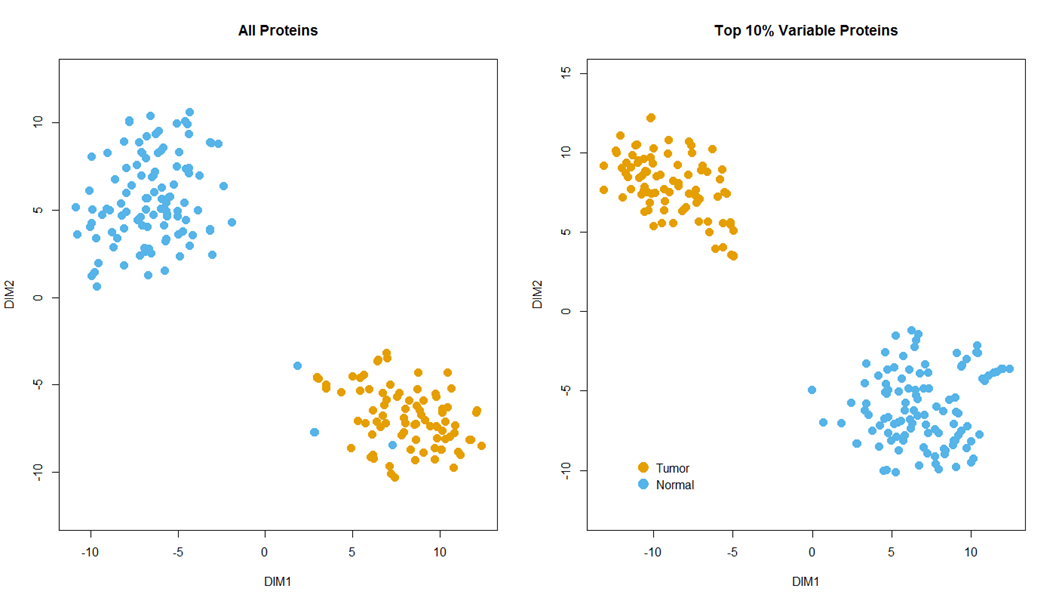
**

**Figure S1**. Unsupervised exploration of the sample discrimination potential of the CPTAC-CCRCC proteomics dataset. Both plots show 2D maps of the projected transformations generated by the t-SNE algorithm. Left plot was obtained when using the full set of proteins available in the CCRCC dataset (9964 proteins). The right plot was obtained when using the top 10% most variable proteins in the dataset.

| **Correctly predicted normal samples** | **Correctly predicted tumor samples** |
| --- | --- |
| 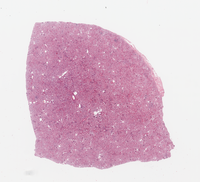 | 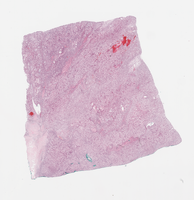 |
| 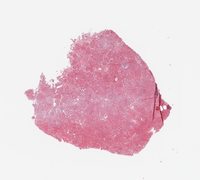 | 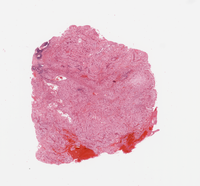 |

**Figure S2**. Examples of normal and tumor samples correctly classified (test dataset) by the DL model.

| **Correctly predicted normal sample** | **Correctly predicted tumor sample** |
| --- | --- |
| 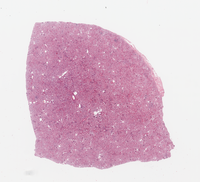 | 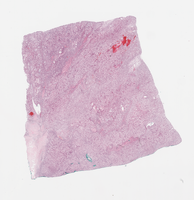 |
| 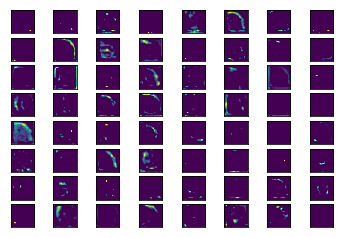 | 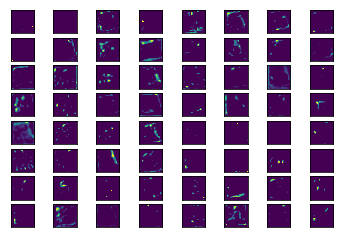 |

**Figure S3.** Examples of normal and tumor samples correctly classified (test dataset) by the DL model and a selection of their corresponding feature maps extracted from the 13^th^ convolutional layer.


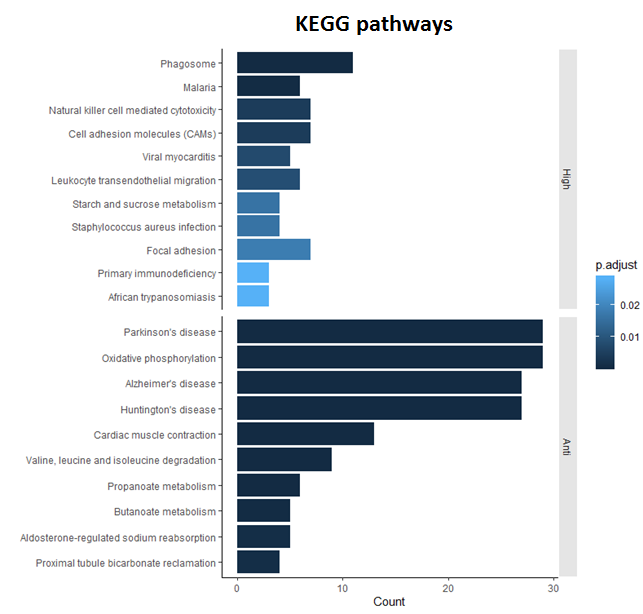


**Figure S4**. KEGG pathway enrichments of the proteins that are either highly positively- or anti-correlated with histology-based predictions. Bars indicate the magnitude of the enrichment scores, and adjusted P-values of the enrichments are color coded.


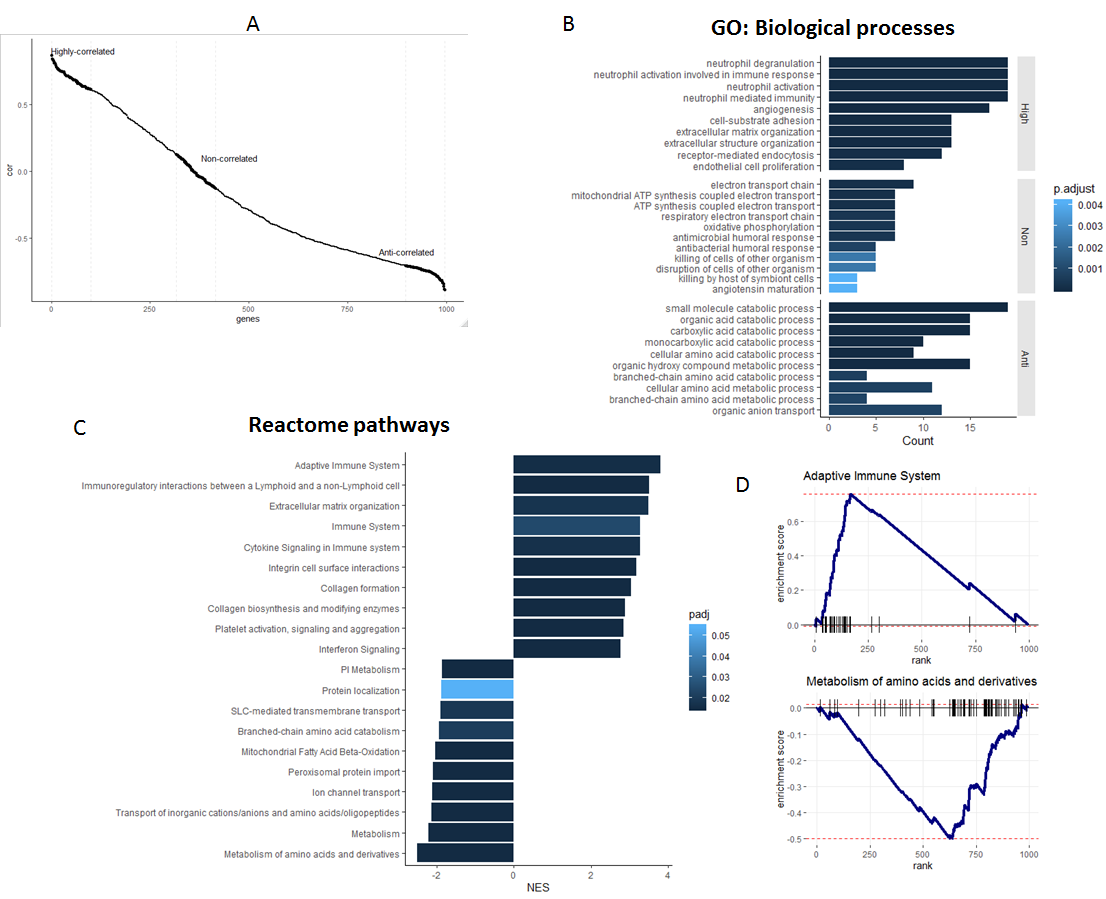


**Figure S5**. Analysis of correlations between RNAs (encoding the diagnostic set of proteins) and the histology-based model predictions. A. Correlation plot of gene expression values and image-based predictions (P-values of assigning an image to the tumor class). B. GO enrichment analysis of proteins highly positively-, anti- and not correlated with histology-based predictions. C. List of Reactome pathways that are significantly associated with the genes on the basis of their correlations with histology-based predictions, as detected by GSEA. D. Examples of pathways significantly associated with proteins highly positively- and anti-correlated with histology-based predictions respectively. In B and C: Bars indicate the magnitude of the enrichment scores, and adjusted P-values of the enrichments are color coded.


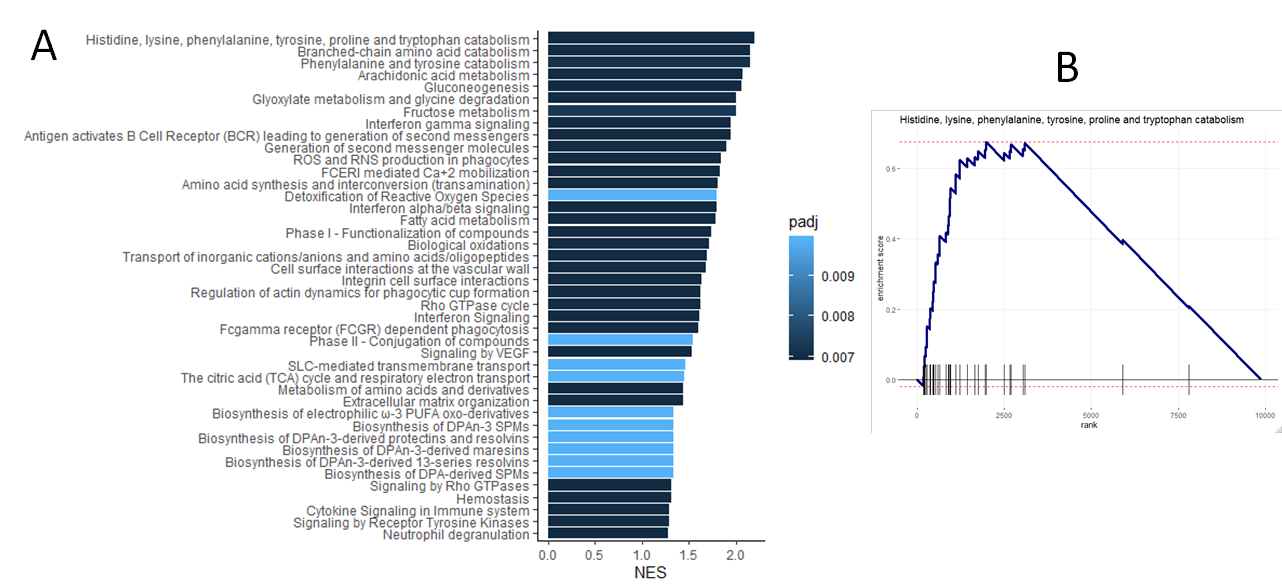


**Figure S6**. Reactome pathways enriched in the full set of 9884 genes available in the CCRCC (RNASeq) dataset. A. List of statistically detected enrichments identified by GSEA. B. Example of a significantly enriched molecular pathway. Genes were ranked on the basis of their expression correlations with their protein expression values, and GSEA estimated pathway enrichment scores. Using the same analysis, no significant enrichments were obtained when focusing on the set of 995 diagnostic proteins.
